# FPGA Implementation for Odor Identification with Depthwise Separable Convolutional Neural Network

**DOI:** 10.3390/s21030832

**Published:** 2021-01-27

**Authors:** Zhuofeng Mo, Dehan Luo, Tengteng Wen, Yu Cheng, Xin Li

**Affiliations:** School of Information Engineering, Guangdong University of Technology, Guangzhou 510006, China; mozf@mail2.gdut.edu.cn (Z.M.); dehanluo@gdut.edu.cn (D.L.); chengyu@gdut.edu.cn (Y.C.); lixin@mail2.gdut.edu.cn (X.L.)

**Keywords:** electronic nose, odor identification, depthwise separable convolutional neural network, FPGA-implementation

## Abstract

The integrated electronic nose (e-nose) design, which integrates sensor arrays and recognition algorithms, has been widely used in different fields. However, the current integrated e-nose system usually suffers from the problem of low accuracy with simple algorithm structure and slow speed with complex algorithm structure. In this article, we propose a method for implementing a deep neural network for odor identification in a small-scale Field-Programmable Gate Array (FPGA). First, a lightweight odor identification with depthwise separable convolutional neural network (OI-DSCNN) is proposed to reduce parameters and accelerate hardware implementation performance. Next, the OI-DSCNN is implemented in a Zynq-7020 SoC chip based on the quantization method, namely, the saturation-flooring KL divergence scheme (SF-KL). The OI-DSCNN was conducted on the Chinese herbal medicine dataset, and simulation experiments and hardware implementation validate its effectiveness. These findings shed light on quick and accurate odor identification in the FPGA.

## 1. Introduction

An electronic nose (e-nose), which mimics biological olfaction, is an odor analysis device composed of a carefully selected sensor array and an appropriate pattern recognition algorithm [1]. E-nose has the ability to detect and distinguishing characteristics such as the type and concentration of gas. E-nose has become more and more widely used in the world. Sigfredo et al. designed an array of gas sensor and five machine learning algorithms to detect and evaluate contamination in grapevine berries and taint in wines [2]. Hao Wei et al. used PEN3 e-nose for data collection and designed a back-propagation neural network (BPNN) to detect brown core in the Chinese pear variety huangguan [3]. Winston Li et al. used four classifiers—MLP, SVM, KNN, and Parzen—and fusion in Dempster–Shafer to improve the accuracy of odor classification [4]. In addition, fluctuation enhanced sensing (FES) has been applied to the field of e-noses to detect gas-phase chemicals [5,6,7]. In FES, noise is considered to carry much useful information, so it uses microfluctuations caused by the interaction between chemical sensors and gas molecules to improve sensitivity and selectivity [8]. With the development of e-nose technology, e-nose has been designed and optimized many times and widely used in food testing [9], environmental monitoring [10,11], medical diagnosis [12,13,14], and the space shuttle [15,16,17]. In particular, for a long time, Chinese herbal medicine has been classified based on traditional identifying methods such as human smell, taste, vision, and touch, while the human smell is the most common method to distinguish the variety and various herb-growing areas. However, human identification of Chinese herbal medicines is highly subjective and time-consuming because individual differences and external disturbances may easily influence humans distinguishing. In recent years, several new technologies have emerged to replace manual classification of Chinese medicinal materials, such as gas chromatography-mass spectrometry (GC–MS) [18] and Electronic tongue (E-tongue) [19]. They are challenging to be commonly used because of their high price or damage to the integrity of Chinese medicinal materials.

At present, many deep learning methods and applications have been proposed, and they were also applied to odor identifications. Danli Wu et al. used a convolutional neural network (CNN) to predict odor pleasantness [20]. You Wang et al. designed an optimized deep convolutional neural network to classify dendrobium data collected by e-nose [21]. Yan Shi et al. designed a CNN-SVM model to classify beer data collected by PEN3 e-nose [22]. Deep learning algorithm can automatically extract and recognize odor features from odor data. Compared with traditional machine learning methods [23,24], the deep learning method has more advantages when applied in the field of odor recognition and achieved better performance.

An e-nose is usually operating in a separate way where the gas sensing hardware collects response data then transmits them to a computer for identification. Considering the feature of real-time data processing, some studies integrated odor identification function into automatic odor sampling hardware. Zhiyuan Wu et al. designed a low-cost e-nose system and identified cigarettes using random forest [10]. A. Ali et al. proposed a hardware/software co-design approach using the Zynq platform based on principal component analysis [25]. The integration of the e-nose system has several advantages. First of all, the integration makes the system less complicated and increase mobility because the identification algorithm runs locally and avoid using an additional computer. Furthermore, integration reduces data transmission, which makes it easier for real-time odor identification. It can be seen that the integrated e-nose design has become an important research direction. Although the integrated e-nose has many benefits, such as portability, low cost, and miniaturization, there are still some challenges in designing the integrated e-nose. The design of integrated e-nose is limited by chip performance, which leads to simple algorithms can be conducted. A simple algorithm may have a certain effect in some specific applications, but the effect may worsen in a complex odor recognition field. Complex algorithms are limited by the computational performance of simple chips, resulting in slow recognition speed, which leads to many excellent deep learning models that usually need to run on computers with GPUs. Therefore, it is vital to contrive this system capable of low-cost, fast identification, and maintaining the accuracy of identification, especially in the practical application of e-nose.

The main contributions of this paper are as follows. (1) Deep separable convolution is applied in odor identification. We propose a lightweight deep learning odor identification model named OI-DSCNN, which balances the speed and accuracy of the odor identification algorithm. (2) Accelerated deep learning algorithm based on Field-Programmable Gate Array (FPGA) is introduced into odor identification, in which the overall architecture and modules of OI-DSCNN are designed and optimized. Therefore, odor identification is accelerated in FPGA. (3) The SF-KL quantization scheme is designed to reduce FPGA resource consumption and maintain the accuracy.

This article is composed as follows. In Section 2, the OI-DSCNN model is first introduced, and then the design and implementation of SF-KL are introduced in detail. In Section 3, the architecture of OI-DSCNN in FPGA, and the design and optimization of each module are illustrated. In Section 4, experiments for the evaluation of the model are demonstrated. In Section 5, conclusions are drawn and future research is prospected.

## 2. The OI-DSCNN Model

### 2.1. Depthwise Separable Convolution

The depthwise separable convolution, initially introduced by L. Sifre et al. [26], requires fewer parameters to reduce the high computational burden of standard convolution. It had been applied to frameworks such as Mobilenet [27,28,29], and it remained sufficiently decent results and performances.

**Figure 1:**
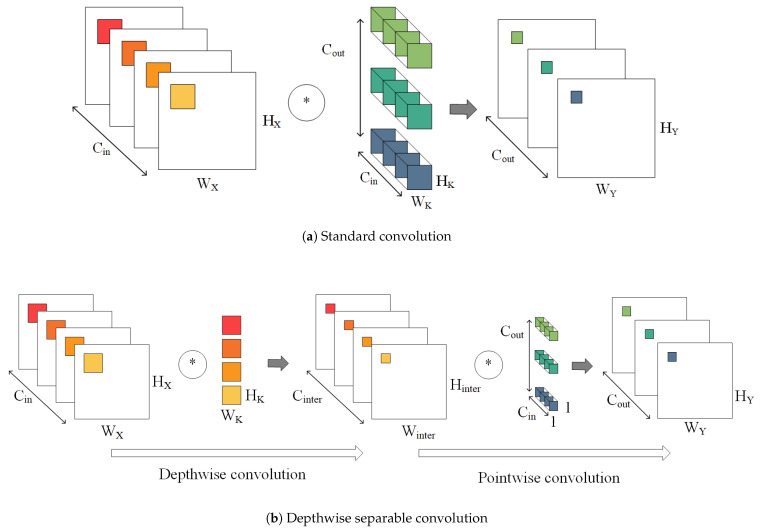
The working principle of standard convolution and depthwise seperable convolution. In depthwise convolution, each convolution kernels is convoluted with each input channels. In pointwise convolution, convolution kernel is a 1×1 standard convolution.

A depthwise separable convolution decomposes a standard convolution into a depthwise convolution and a pointwise convolution. Figure 1 shows the working principle of a standard convolution and depthwise separable convolution.

As for standard convolutions, as shown in Figure 1a, each input channel requires a convolution that the number of convolution kernels is the same as the output channel. The result of each output channels is the sum of its corresponding convolution kernels and convolutional results of all input channels. Suppose that the size of the input *X* is $W_{X}\cdot H_{X}\cdot C_{in}$, where $W_{X}$, $H_{X}$, and $C_{in}$ is the width, height, and the number of input channels, respectively. The output *Y* is $W_{Y}\cdot H_{Y}\cdot C_{out}$, where $W_{Y}$, $H_{Y}$, and $C_{out}$ are the width, height, and the number of output channels, respectively. Compared with the standard convolution, depthwise separable convolution separates the network into depthwise convolution and pointwise convolution, which reduces the number of weights and the amount of computation:

(1) $$\frac{N_{K_{DC}}+N_{K_{PC}}}{N_{K_{SC}}}=\frac{W_{K}\cdot H_{K}\cdot C_{inter}+C_{inter}\cdot C_{out}}{W_{K}\cdot H_{K}\cdot C_{in}\cdot C_{out}}=\frac{1}{C_{in}}+\frac{1}{W_{K}\cdot H_{K}}$$

where $N_{K_{SC}}$ is the size of the standard convolution kernels. $N_{K_{DC}}$ is the size of the depthwise convolution kernels. $N_{K_{PC}}$ is the size of the pointwise convolution kernels. $C_{inter}$ is intermediate channels of depthwise separable convolutional network.

### 2.2. OI-DSCNN Modeling

**Figure 2:**
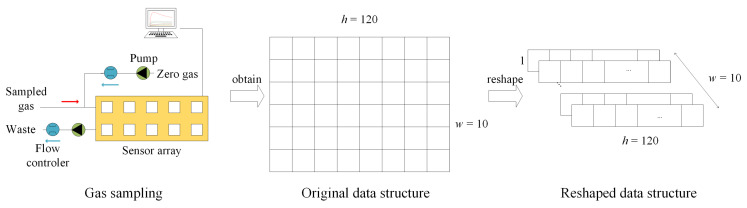
Input data from PEN3 e-nose.

The 2-dimensional sensing responses collected from e-noses were used directly in many studies [20,30,31,32,33,34,35]. In our studies, the w×h input data were reshaped to w×1×h for depthwise convolution computations of *w* independent channels as shown in Figure 2.

**Figure 3:**
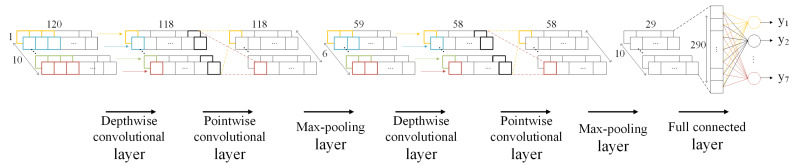
The OI-DSCNN model.

The structure of the OI-DSCNN model is shown in Figure 3 and Table 1. It consists of two depthwise separable convolution layers and a fully connected layer, each depthwise separable convolution layer with a max-pooling layer. Softmax classifier is used for the identification. The two depthwise convolution layers consisted of 1×3 and 1×2 convolution kernels, respectively. Nonlinear activation function Relu is selected in all convolutional layers. The stride size of all convolutional layers is set to 1, and the max-pooling layer is set 2.

### 2.3. Quantization

Generally speaking, a 32-bit floating-point arithmetic consumes four times more DSPs than a 8-bit fixed-point arithmetic in parallel operation, and it requires over two times computation timing. Besides, it consumes more block memory (BRAM) and registers resources to store a large amount of parameters and intermediate computing results. To decrease the number of resources used, currently, many effective quantization methods have been proposed [36,37,38], which these methods were mostly designed for GPUs and CPUs but not for FPGA. Some common methods can decrease the model accuracy under the same network scale in the quantization phase in FPGA [39,40]. Here, we proposed a saturation-flooring KL_divergence scheme (SF-KL) to reduce the usage of computing devices because of the limited DSP resource in the FPGA chip.

The OI-DSCNN is first trained and validated in floating-point operation, then the proposed SF-KL is used to find the optimized saturation-flooring bits of outputs on each layers. After that, the quantization methods were designed in hardware logic. There are three parts of quantization process as follows.

#### 2.3.1. Quantization of Model Weights

The OI-DSCNN model is fully trained using floating-point algorithms. After the training, parameters were first converted from 32-bit floating-point to 8-bit fixed-point using linear quantization. The following is the procedure of how to quantize weights.

We need to find the maximum weight in each layer:

(2) $$M_{i}=\max\left(\mathrm{abs}\left(P_{i}\right)\right),$$

where i=1,…,m, *m* is the number of layers in the model. $P_{i}$ represents weights in each layer:

(3) $$P_{i}=\left[\begin{array}{cccc} p_{1} & p_{2} & \ldots & p_{n} \end{array}\right]$$

where *n* is the number of weights in a layer.

The scale factor sf is obtained by

(4) $$sf_{i}=\frac{2^{8}-1}{M_{i}}$$

The quantized value $Q_{P_{i}}$ of $P_{i}$ is:

(5) $$Q_{P_{i}}=\mathrm{round}\left(P_{i}\cdot sf_{i}\right)$$

#### 2.3.2. Quantization of Input

As the different sensitivities and selectivities to various gases on gas sensors, the input was mean normalized to [−1,1]:

(6) $$\mu_{i}=\frac{\sum_{j}x_{i,j}}{120}$$

(7) $$x_{i,j}^{\prime }=\frac{x_{i,j}-\mu_{i}}{\max\left(x_{i}\right)-\min\left(x_{i}\right)}$$

where j=1,2,…,120,i=1,2,…,10. $\mu_{i}$, $\max(x_{i})$ and $\min(x_{i})$ are the average value, maximum value, and minimum value of sampling points, respectively. $x_{i,j}$ and $x_{i,j}^{\prime }$ represent the original input and normalized input, respectively. After that, the input is linearly quantized to the N-bit fixed-point number $Q_{x_{i,j}}$:

(8) $$Q_{x_{i,j}}=\mathrm{floor}\left(x_{i,j}^{\prime }\cdot \left(2^{8}-1\right)\right)$$

#### 2.3.3. Quantization of Output on Each Layer

In order to reduce the workload of manual attempts and avoid introducing additional parameters, an output quantization is shown in Equation (9). It calculates by the floor and saturation operating, which the scale factor is constrained to $\frac{1}{2^{N}}$:

(9) $$Q_{Y_{N}}=\left\{\begin{array}{ccc} \; & \;\;\;\;\;127 & \frac{Y}{2^{N}}\geq 127\\ \; & \mathrm{floor}(\frac{Y}{2^{N}}) & -128<\frac{Y}{2^{N}}<127\\ \; & \;\;-128 & \frac{Y}{2^{N}}\leq -128 \end{array}\right.$$

where *Y* is the output, and $Q_{Y_{N}}$ is the quantized output. In this way, the quantization of the output can be operated only by floor and saturation without multipliers, thereby reducing the consumption of DSPs. Algorithm 1 demonstrated the completed procedure of SF-KL obtaining the scale factor.
**Algorithm 1** SF-KL scheme**Input:**$X_{n}$;$Q_{X_{n}}$; *P*; $Q_{P}$ **Output:**$sf_{i}$1:/*define Model A and B, while A uses floating-point and B uses integer*/2:A,B=OI−DSCNN(Weight,Data)3:**function**Quantization($X_{n}$, $Q_{X_{n}}$, *P*, $Q_{P}$)4:    /*j=DWC1,PWC1,…,FC*/5:    **for** *j* **in** OI−DSCNN **do**6:        **for** k=1→n **do**7:           **if** j≠Maxpooling **then**8:               /* $X_{k}^{\prime }$ are the input for next layer*/9:               $X_{k}^{\prime }=A.j\left(P_{j},X_{k}\right)$10:               $Y_{k}=B.j\left(Q_{P_{j}},Q_{X_{n}}\right)$11:               /*max = the highest bit of the output value - 8*/12:               **for** N=0→max **do**13:                   /*KL_divergence*/14:                   $KL_{N_{k}}\left(X_{k}^{\prime },Q_{Y_{N_{k}}}\right)$15:               **end for**16:           **end if**17:        **end for**18:        $KL_{N}=\sum_{k}KL_{N_{k}}$19:        /*Find the minimum KL_divergence and get N*/20:        $N\leftarrow min(KL_{N})$21:        /*Get the scale factor*/22:        $sf_{j}\leftarrow \frac{1}{2^{N}}$23:    **end for**24:**end function**

First, it needs to prepare the original weight of *P* and the quantized weight $Q_{P}$. *n* original training samples $X_{n}$ were randomly selected to calculate scale factor $\frac{1}{2^{N}}$. The selected samples $X_{n}$ were quantized to $Q_{X_{n}}$ according to Section 2.3.2. Three samples of each type are randomly selected, which a total of n=3×7=21 samples as input to the algorithm. Two identical OI-DSCNN models are constructed. The difference between the two models is that one uses floating-point calculations and the other uses integer calculations. Then, import the samples into the two models separately. The results calculated by each layer in the two models (except for maximum pooling) will be used to calculate KL_divergence:

(10) $$KL\_divergence\left(X_{k}^{\prime },Q_{Y_{N}}\right)=\mathrm{sum}\left(X_{k}^{\prime }\cdot \log\left(\frac{X_{k}^{\prime }}{Q_{Y_{N}}}\right)\right)$$

$X_{k}^{\prime }$ is the output of floating point, and $Q_{Y_{N}}$ is the output of integer number after saturation-flooring. Then, the scale factor of the smallest value of KL_divergence is used as the input of the next layer of integer number. We use the minimum value of the mean of the KL_divergence of different scale factors due to the scale factor. As a nonlinear Relu activation function is used, for the n-bit output Y[n−1:0] in FPGA, the Equation (9) is transformed as

(11) $$Q_{Y_{N}}=\left\{\begin{array}{cc} 8^{\prime }b0 & \mathrm{if}\;Y[n-1]=1\\ \left\{0,Y[N+6:N]\right\} & \mathrm{elseif}\;(|Y[n-1:N+7])=0\\ \left\{0,7^{\prime }b111\_1111\right\} & \mathrm{other}\; \end{array}\right.$$

In this way, it quantizes the output of each layer to 8 bits so that it can reduce the input bitwidth of the following layers.

## 3. FPGA Design and Implementation

**Figure 4:**
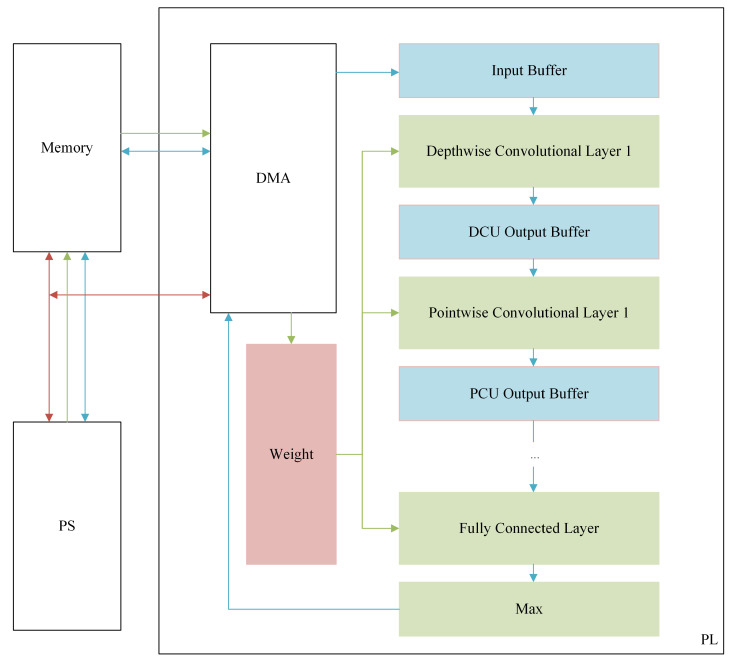
An overview of the FPGA implementation architecture of OI-DSCNN. The red line is the control path. The green line is the weight path. The blue line is the data path.

Figure 4 describes the overview of the system architecture. This system was implemented on the Digilent Arty Z7-7020 development board, USA, which is detailed in Section 4. In order to take advantage of parallel computation in FPGA, the four convolutional layers and the fully connected layer are operated in a pipeline. In order to reduce the data access time and improve the performance of the system, the output of each layer of the network is stored in on-chip memory. The system is composed of PS block, PL block, and DDR memory. The PL block composes of winograd depthwise convolution unit (WDCU), pointwise convolution unit (PCU), fully connected unit (FCU), on-chip memory, and direct memory access (DMA). WDCU, PCU, and FCU are responsible for calculating each convolutional layer, activation layer, max-pooling layer, and fully connected layer. On-chip memory includes input data buffer, intermediate data buffer, and weight buffer. The input data buffer, intermediate data buffer, and the weight buffer for the fully connected layer were designed by block RAM (BRAM), while the weights of convolutional layers are stored in distributed RAM (DRAM). Thank to some techniques used when modeling OI-DSCNN, the number of parameters and computational complexity saw a significant decrease, which the experimental results will be illustrated in detail in Section 4. Besides, the BRAM with 630 KB in a Zynq-7020 chip is sufficient to store such a small scale of several input samples when the model is implemented in the pipeline. Moreover, it is also sufficient to store intermediate results, input data, and parameters in the on-chip memory, which reduces the memory access timing and enhances the performance of system.

The core of the PL design is the convolution layer, which significantly impacts the computation rate of the entire system. Due to the grouped convolution structure of the depthwise convolution layer, the data within each input channel cannot be reused. The Winograd algorithm for depthwise convolution was introduced [41], and the structure of the WDCU was presented in Section S1. WDCU consists of line buffer, input transformation unit, multiplier array unit, configurable output inverse transformation unit, and relu and quantization unit. A line buffer is used to buffer the data transferred to the input transformation unit. The transformed input data are transmitted to the multiplier array unit that multiplies the inputs and the weights from the WDCU weight buffer unit. After the inverse transformation of the Winograd algorithm is completed by the configurable output inverse transformation unit, the 8-bit output of the depthwise convolutional layer is obtained through relu and quantization unit and transmitted to the WDCU output buffer.

The PCU, which is used for pointwise convolution, comprises line buffer, multiplier array unit, adder tree unit, relu and quantization unit, and maxpooling unit. The output of WDCU is connected to the line buffer of the first PCU, then passed to the line buffer of the next PCU. Details about the design of PCU can be seen in Section S2. For the fully connected layer, as the weights and data are not reused, a compromise solution is adopted in our design [42,43]. The FCU, which is used to calculate the output of fully connected layer, comprises line buffer, multiplier array unit, adder tree unit, and accumulator unit. FCU divides the data from the PCU output buffer into several 1×5 small scale vectors and obtains the output through multiplier array unit, adder tree unit, accumulator unit, and quantization unit. Details about the design of FCU can be seen in Section S3. The final outputs of FCU are compared in the max unit, and then the comparison result is sent to the PS block.

## 4. Experiments and Results

### 4.1. Experimental Setup

The data set was collected from an e-nose, PEN3, AIRSENSE analytics Inc, Germany. PEN3 e-nose is a general gas response signal sampling instrument, which has 10 metal oxide gas sensors, and each sensor has a different sensitivity to different gases, as shown in Table 2. Thanks to a sensor array composed of ten gas sensors, PEN-3 has the ability to sense various gases. The settings of the e-nose are described in Table 3.

PEN3 was used to collect gas sensing responses of seven Chinese medicinal materials as dataset: betel, galangal, fructus amomi, fructus aurantii, curcuma zedoary, rhizoma zingiberis, and moutan bark. The experimental environment temperature of the e-nose was $25\pm 0.5{\;}^{\circ }$C, and the humidity was 75±2%. The procedures for preparing these materials were set as follows.

**Figure 5:**
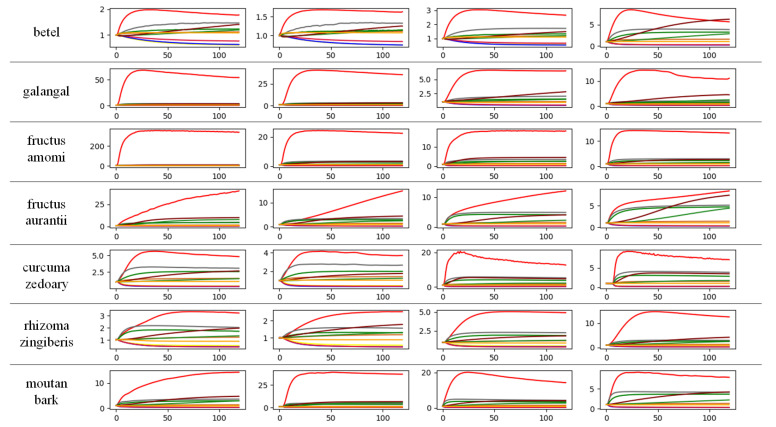
Part of Chinese herbal medicines dataset.

1. The materials were placed in a clean beaker and kept still for more than 20 min.
2. Before collecting the data, the sensor chamber is cleaned and calibrated.
3. Data collection was conducted, and each sample was collected for 120 s.
4. Steps 1–3 were repeated, and 100 samples of each kind of Chinese herbal medicine were collected. The final dataset consisted of 700 data samples in 7 categories, 100 samples for each category, as shown in the Figure 5.

We compared the OI-DSCNN model with several odor identification model methods. We mainly used PyTorch and scikit-learn in Python to model these algorithms. Pytorch is a deep learning framework for Python [44]. Scikit-learn is a Python module integrating a wide range of state-of-the-art machine learning algorithms [45].

Stochastic Gradient Descent (SGD) was used to train the model. The number of samples per training was 21, and the momentum was 0.9. The learning rate was initially set to 0.01 and adjusted by ReduceLROnPlateau(). When the loss of validation set has stopped improving, it was divided by 10 and finally stopped at 0.0001. The loss function was set to CrossEntropyLoss().

The design was coded in Verilog HDL and synthesized and implemented on Xilinx Vivado. The OI-DSCNN model was evaluated on a Digilent Arty Z7-7020 development board. The Arty Z7-7020 development board contained a Zynq-7020 FPGA chip, a Zynq-7000TM All Programmable System-on-chip (AP SoC). The AP SoC consists of the Programmable Logic (PL) block and the Processing System (PS) block. The Arty Z7-7020 platform characteristics are shown in Table 4. Three different types of platforms, that is, Raspberry Pi 4B, CPU, and GPU with similar or slightly better performances, were selected to compare the performance with FPGA.

### 4.2. Results

**Figure 6:**
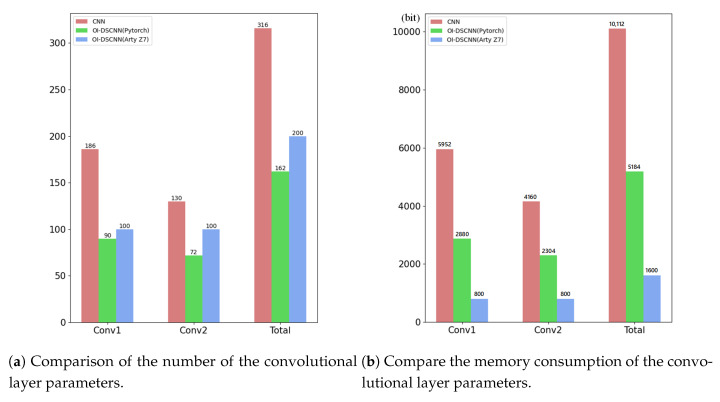
Convolutional layer parameters for CNN, OI-DSCNN in Pytorch, and OI-DSCNN in Arty Z7.

OI-DSCNN has the advantage of less convolution kernel parameters. To evaluate this, CNN was modeled to compare the number of used parameters. The architecture of CNN was shown in Table 5. As Figure 6a displayed, the total amount of convolutional layer parameters between CNN and OI-DSCNN dropped dramatically from 316 to 162 in Pytorch. In Arty Z7, the Winograd algorithm was used, and bias was removed because of the computing acceleration and better performance. OI-DSCNN has a slight increase in the total amount of convolutional layer parameters between the PC and FPGA platforms. The convolutional layer parameters of OI-DSCNN are quantized as 8-bit fixed-point numbers when eventually implemented in FPGA. The OI-DSCNN requires less memory storage resource as shown in Figure 6b. It can be noticed that the convolutional layer parameters of an OIDSCNN requires 1600 bits of memory, where a CNN requires 10,112 bits. The amount of memory used in OI-DSCNN has a significant reduction to 15.8% of the CNN model.

Several algorithms were built for comparison to evaluate the effectiveness of OI-DSCNN. Fivefold cross-validation was used to verify the accuracy of each model in the PC. Simultaneously, OI-DSCNN was quantized and run in Arty Z7 during the cross-validation process to verify the performance of the model on the FPGA. As shown in the Table 6, CNN had the best performance among these models where it took the average score of 0.9457. OI-DSCNN in floating-point calculation had the second-best performance, which was 0.9414, while it had the lowest score of 0.2443 when it operated in 8-bit fixed-point arithmetic. The OI-DSCNN, without bias, operated in 8-bit fixed-point arithmetic had a decent score of 0.9371. Although the accuracy score OI-DSCNN had a narrowed gap between CNN, CNN+SVM [22], and OI-DSCNN in floating-point arithmetic, OI-DSCNN requires less memory and DSP resources in parallel kernel-operations. Decision tree (DT), multilayer perception (MLP) [46], and principal component analysis with decision tree (PCA+DT) [25] had scores of 0.25, 0.7757, and 0.2686, respectively.

**Figure 7:**
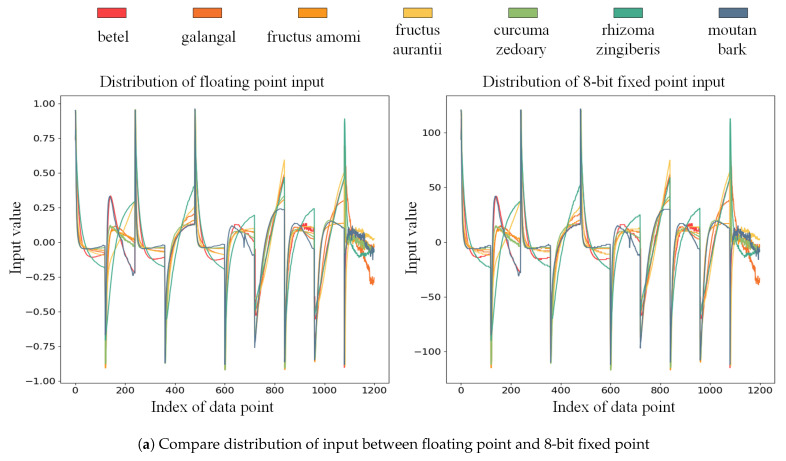

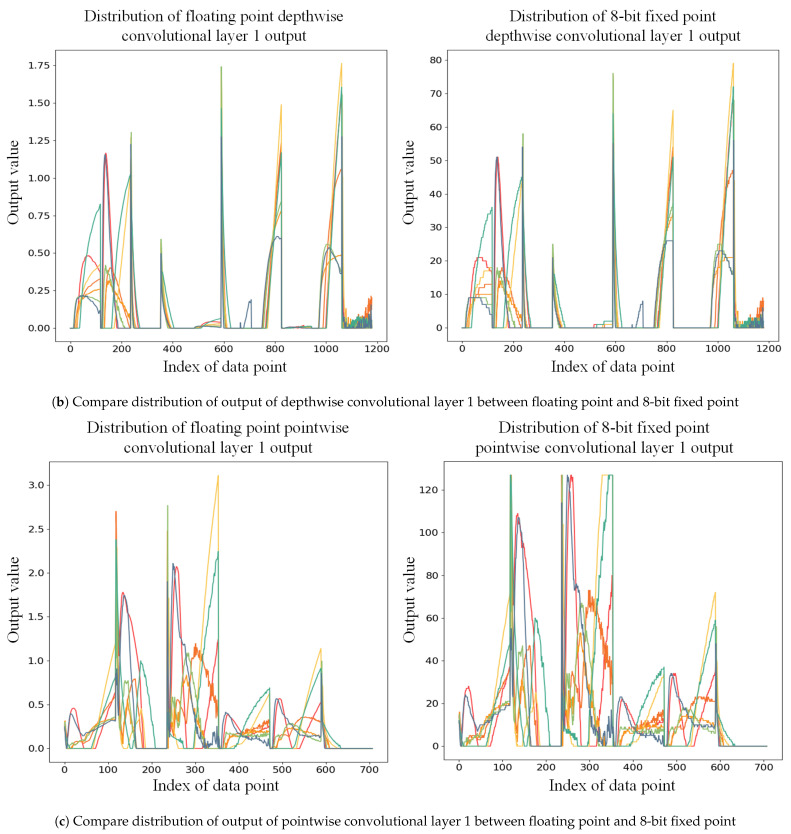

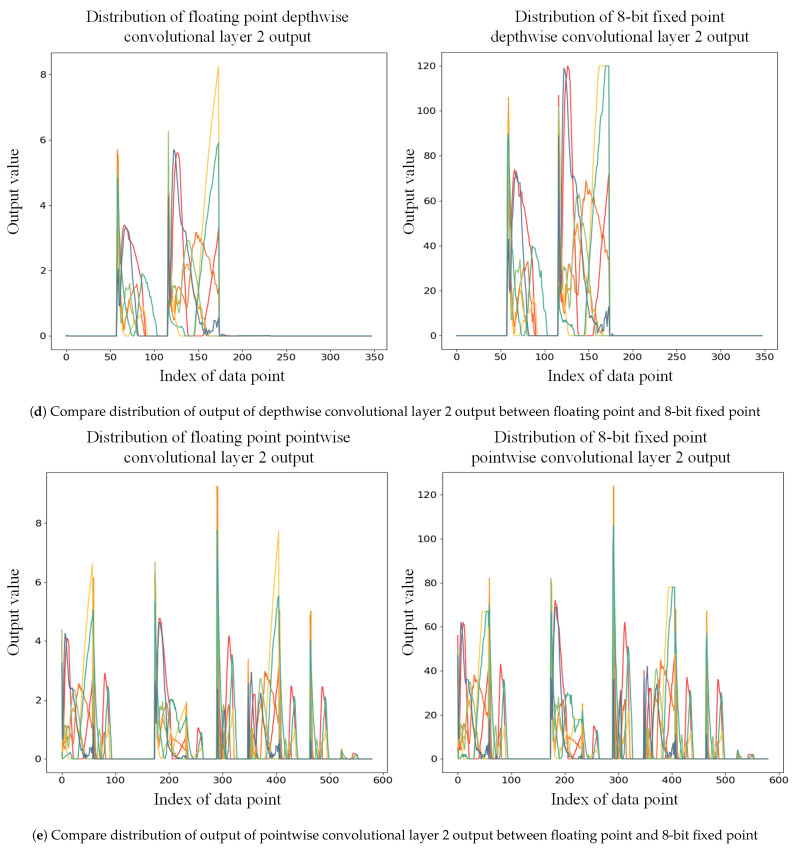

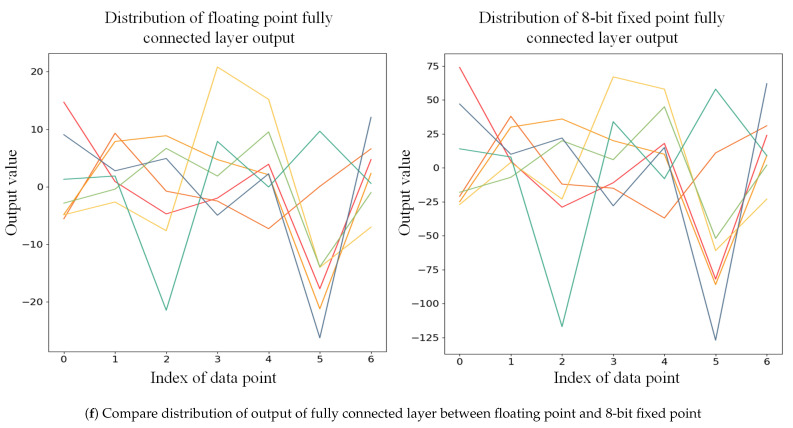
The input and output distribution of 7 classes of Chinese medicinal materials in OI-DSCNN. The left side of each subfigure is the distribution of OI-DSCNN at the floating point. The right side of each subfigure is the distribution of OI-DSCNN at an 8-bit fixed point.

As the bit width of the output on WDCU, PCU, and FCU blocks are 20-bit, 22-bit, and 22-bit signed fixed points, respectively, the goal of each quantization unit in WDCU, PCU, and FCU is to intercept 8 bits of the result as the final output. Seven samples for each medicine were randomly selected to overview the effectiveness of quantization, as shown in Figure 7. The feature map is reshaped into a one-dimensional matrix for visualization. It can be seen from Figure 7a that the input can maintain a similar distribution after being quantized from floating-point to 8-bit fixed point. It can be noticed that the quantized output of saturation and flooring will losses some information because some lower bits are cut off, and some tiny spikes may be cut off because of the saturation, as shown in Figure 7b–f. However, there is not much difference between the distribution of the actual output and the quantized output distribution. This quantization method does not require any calculations during the implementation period, as long as the saturation cut off and flooring are performed, which is very suitable for FPGAs.

OI-DSCNN in 8-bit fixed point operating utilizes less computing resources which can be implemented in some low-cost FPGA platforms. Table 7 demonstrated the resource utilization of OI-DSCNN in the Arty z7-7020 platform. On such a restricted DSP-resource the platform, the algorithm was successfully implemented. A few slice look-up tables (LUTs), registers, and BRAM resources have been consumed, which can be used by the remaining resources to implement other functions.

Finally, a crosswise comparison in commonly used platforms was conducted. Table 8 illustrated the performance comparison of OI-DSCNN on Raspberry Pi 4B which is made in UK Raspberry Pi foundation, CPU, GPU, and Arty z7-7020 platforms. The frequency of the system clock in the Arty Z7-7020 platform was 100MHz. We collected CPU and GPU runtime from PyTorch functions profiler. A random sample in the test set was used to test the time for each platform to run OI-DSCNN inference. The model implemented in the Arty platform had a noticeable advance compared to other platforms, where it was 38 times faster than computing in i7-10875H. After that, test set was used to evaluate the average inference time of each platform. Each odor processing time on Arty Z7 is 20.8 μs, which was better than other platforms. It can be seen that GPU is suitable for training using batch processing models but not for the inference process, especially when the odor data set is small.

## 5. Conclusions

In this paper, a lightweight OI-DSCNN is proposed for odor identification. The implementation of separable depthwise convolution and the Winograd algorithm could reduce the number of convolution parameters and accelerate the odor identifying rate. Additionally, the SF-KL is designed to quantize the outputs of each layer of OI-DSCNN and maintain high accuracy. Finally, the OI-DSCNN is successfully implemented in Zynq-7020. The experimental result demonstrates the effectiveness of the system. In summary, OI-DSCNN implemented in FPGA contains fewer parameters and runs faster with higher accuracy. The integration of odor identification algorithms on odor collection devices will become a trend for designing lighter and real-time processing e-noses. Therefore, the integration of e-nose with the balance of performance and cost must be taken into account. Some techniques may be considered to optimize the model, such as better quantization methods and specific parameter reduction strategies. Moreover, FES, a technology that obtains more information from sensors, can be applied to our future research.

## Figures and Tables

**Table 1:** the OI-DSCNN model architecture.

Layer	Type	Filter Shape	Input Size
Depthwise separable convolution 1	Depthwise convolution	1∗3∗10	1∗120∗10
	Pointwise convolution	1∗1∗10∗6	1∗118∗10
	Max-pooling	1∗2	1∗118∗6
Depthwise separable convolution 2	Depthwise convolution	1∗2∗6	1∗59∗6
	Pointwise convolution	1∗1∗6∗10	1∗58∗6
	Max-pooling	1∗2	1∗58∗10
Fully connected 3	Fully Connected	290∗7	1∗29∗10
Classifier	Softmax	-	7

**Table 2:** Sensor array details in PEN-3 e-nose.

Sensor	Sensor Sensitivity and General Description
W1C	Aromatic compounds.
W5S	Very sensitive, broad range of sensitivity, reacts to nitrogen oxides, very sensitive with negative signals.
W3C	Ammonia, used as sensor for aromatic compounds.
W6S	Mainly hydrogen.
W5C	Alkanes, aromatic compounds, less polar compounds.
W1S	Sensitive to methane. Broad range.
W1W	Reacts to sulfur compounds, H2S. Otherwise sensitive to many terpenes and sulfur-containing organic compounds.
W2S	Detects alcohol, partially aromatic compounds, broad range.
W2W	Aromatic compounds, sulfur organic compounds.
W3S	Reacts to high concentrations (>100 mg/kg) of methane–aliphatic compounds.

**Table 3:** Settings of PEN-3 e-nose.

Options	Settings
Sample interval	1.0 s
Presampling time	5.0 s
Zero point trim time	5.0 s
Measurement time	120 s
Flushing time	120 s
Chamber flow	150 mL/min
Initial injection flow	150 mL/min

**Table 4:** Some specifications of Arty Z7-7020 development board.

Item	Specification
Product variant	Arty Z7-7020
Zynq part	XC7Z020-1CLG400C
Look-up Tables (LUTs)	53,200
Flip-Flops	106,400
Block RAM	630 KB
DSPs	220
Clock Management Tiles	4
Processor	650 MHz dual-core Cortex-A9 processor 512 MB DDR3 with 16-bit bus @ 1050 Mbps
Memory	16 MB Quad-SPI Flash with factory programmed 48-bit globally unique microSD slot
expansion connectors	Two standard Pmod ports Arduino/chipKIT Shield connector

**Table 5:** The CNN model architecture.

Layer	Type	Filter Shape	Input Size
Convolution 1	Standard convolution	10∗3∗6	10∗120∗1
	Max-pooling	1∗2	1∗118∗6
Convolution 2	Standard convolution	1∗2∗6∗10	1∗59∗6
	Max-pooling	1∗2	1∗58∗10
Fully connected 3	Fully connected	290∗7	1∗29∗10
Classifier	Softmax	-	7

**Table 6:** Model performance comparison.

Model	Platform	5-Fold Accuracy					
		1	2	3	4	5	Average
MLP	PC	0.1143	0.2929	0.1643	0.2643	0.4143	0.25
DT	PC	0.65	0.8714	0.7571	0.8071	0.7929	0.7757
PCA + DT	PC	0.3857	0.2929	0.3857	0.1643	0.1143	0.2686
CNN	PC	0.9357	0.95	0.9571	0.9429	0.9429	**0.9457**
CNN + SVM	PC	0.8786	0.8714	0.9214	0.8643	0.8857	0.8843
OI-DSCNN	PC	0.9286	0.9429	0.9643	0.9357	0.9357	0.9414
OI-DSCNN(bias)	Arty Z7	0.2143	0.25	0.2643	0.2357	0.2571	0.2443
OI-DSCNN(nobias)	Arty Z7	0.9286	0.9357	0.9571	0.9357	0.9286	**0.9371**

**Table 7:** Resource utilization of OI-DSCNN on FPGA.

Type	Slice LUTs	Slice Registers	Block RAM Tile	DSPs
buff	784	1957	9.5	0
Depthwise convolutional layer 1	2305	3164	5	40
Pointwise convolutional layer 1	1309	1878	3	60
Depthwise convolutional layer 2	1434	1980	3	24
Pointwise convolutional layer 2	1396	2130	5	60
Fully connected layer	758	1385	0	35
Utilization	7986	12,494	25.5	219
Avaliable	53,200	106,400	140	220
Percent (%)	15	11.74	18.21	99.5

**Table 8:** Comparison between Raspberry Pi, CPU, GPU, and FPGA implementations for OI-DSCNN.

Platform	One Sample Performance		All Sample Performance	
	Time	Speedup	Average Time	Speedup
Raspberry Pi 4B	4.6 ms	0.2×	366.5 μs	0.1×
i7-8750H	6.3 ms	0.3×	57 μs	0.9×
i7-10875H	2.1 ms	1.0×	52 μs	1.0×
NVIDIA GeForce MX250	1107 ms	-	6.325 ms	-
NVIDIA GeForce GTX 2060	910 ms	-	5.2 ms	-
**Arty Z7-7020**	**57.3 μs**	37×	**20.8 μs**	2.5×

## Data Availability

Data sharing not applicable.

## Supplementary Materials

The following are available online at https://www.mdpi.com/1424-8220/21/3/832/s1, Section S1: WDCU Design, Section S2: PCU Design, Section S3: FCU Design.

## Abbreviations

The following abbreviations are used in this manuscript:

FPGA	Field-Programmable Gate Array
OI-DSCNN	Odor Identification with Depthwise Separable Convolutional Neural Network
CNN	Convolutional Neural Network
WDCU	Winograd Depthwise Convolution Unit
PCU	Pointwise Convolution Unit
FCU	Fully Connected Unit
KL_divergence	Kullback-Leibler Divergence
DMA	Direct Memory Access
BRAM	Block RAM
DRAM	Distributed RAM
PL	Programmable Logic
PS	Processing System
