# Supplementary material for: FPGA Implementation for Odor Identification with Depthwise Separable Convolutional Neural Network
Source: Sensors (Basel). 2021 Jan 27;21(3):832. doi: 10.3390/s21030832 (PMC7865880; doi:10.3390/s21030832)
Supplement: Supplementary file 1 [file sensors-21-00832-s001.pdf]

Supplementary Materials

Section S1. WDCU Design

The core of the PL design is the convolution layer, which significantly impacts the computation rate of entire system. Due to the depthwise convolutional layer's grouped convolution structure, the data between each input channel cannot be reused. The winograd algorithm for depthwise convolution was introduced [1], the structure of the WDCU was displayed in Figure S1. In order to speed up the computation rate, 10 entities of WDCU were instantiated, which they are running simultaneously and in parallel on one convolution process. Weight conversion of the Winograd algorithm is conducted after the model is trained, and the conversion results are saved in a DRAM block. The process of the Winograd conversion is described as follows.

As for the first depthwise convolutional layer, the conversion of the 1×3 convolution kernel is:

$$\begin{cases} w_0 = g_0 \\ w_1 = round\left(\frac{g_0 + g_1 + g_2}{2}\right) \\ w_2 = round\left(\frac{g_0 - g_1 + g_2}{2}\right) \\ w_3 = g_2 \end{cases} \tag{S1}$$

As for the second depthwise convolutional layer, the conversion of the 1×2 convolution kernel is:

$$\begin{cases} w_0 = g_0 \\ w_1 = round\left(\frac{g_0 + g_1}{2}\right) \\ w_2 = round\left(\frac{g_0 - g_1}{2}\right) \\ w_3 = g_1 \end{cases} \tag{S2}$$

where g is the original parameter, and w is the transformed parameter.

Line buffer units were used to buffer for input transformation. The input transformation is shown in equation S3. The transformed input data will be transmitted to the Multiplier array unit that implements the input's multiplication and the corresponding weights from the WDCU weight buffer unit.

$$\begin{cases} x_0 = d_0 - d_2 \\ x_1 = d_1 + d_2 \\ x_2 = d_2 - d_1 \\ x_3 = d_3 - d_1 \end{cases} \tag{S3}$$

where d is the input data, and x is the output value of the input transformation unit.

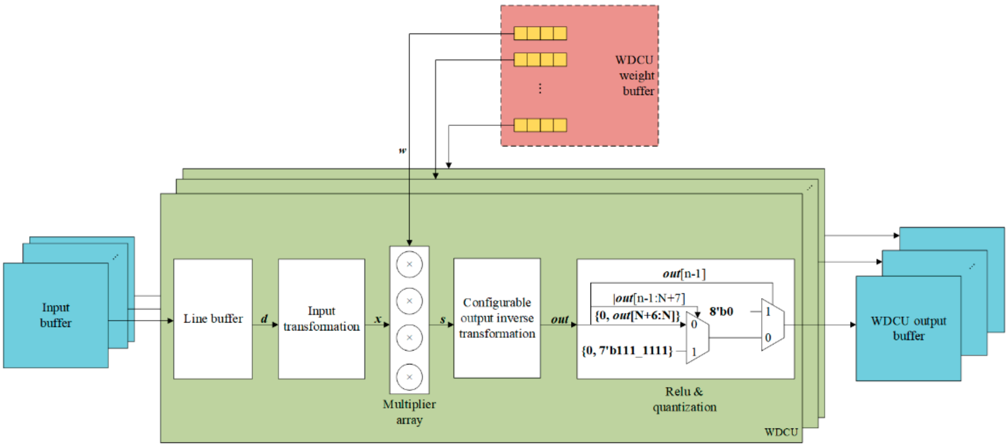

Figure S1. Design of WDCU. The converted weights are stored in DRAM where in WDCU Weight Buffer. The input of the first depthwise convolutional layer is received from an input buffer connected to the DMA block. The input of the second depthwise convolutional layer is received from the first pointwise convolutional layer.

In the OI-DSCNN model, since the size of the convolution kernel of different depthwise convolutional layers is different, a configurable output inverse transformation unit is designed in the WDCU, which supports winograd algorithm $F(2,3)$ and $F(3,2)$ for fast convolution. As for the 1×3 convolution kernel, the configurable output inverse transform unit is illustrated as follows:

$$\begin{cases} out_0 = s_0 + s_1 + s_2 \\ out_1 = s_1 - s_2 + s_3 \end{cases} \quad (S4)$$

As for the 1×2 convolution kernel, the configurable output inverse transformation unit is as follows:

$$\begin{cases} out_0 = s_0 + s_1 + s_2 \\ out_1 = s_1 - s_2 \\ out_2 = s_1 + s_2 + s_3 \end{cases} \quad (S5)$$

where out is the configurable output inverse transform unit's output value, s is the multiplier array unit's output value. The final output of WDCU are quantized to 8-bit signed fixed points in relu-activation & quantization unit as Equation 11 in article.

Section S2. PCU Design

The PCU comprises line buffer, multiplier array unit, adder tree unit, relu unit, and maxpooling unit. As shown in Figure S2, the result of WDCU is connected to the line buffer of the first PCU, then passed to the line buffer of the next PCU, and so on. Each multiplier array unit multiplies data from its line buffer with its corresponding weights stored in the PCU weight buffer unit, and then each adder tree unit adds its multiplication results. After that, a relu-activation & quantization unit and a max-pooling unit are used in each PCU. Then the final output is sent to the PCU output buffers.

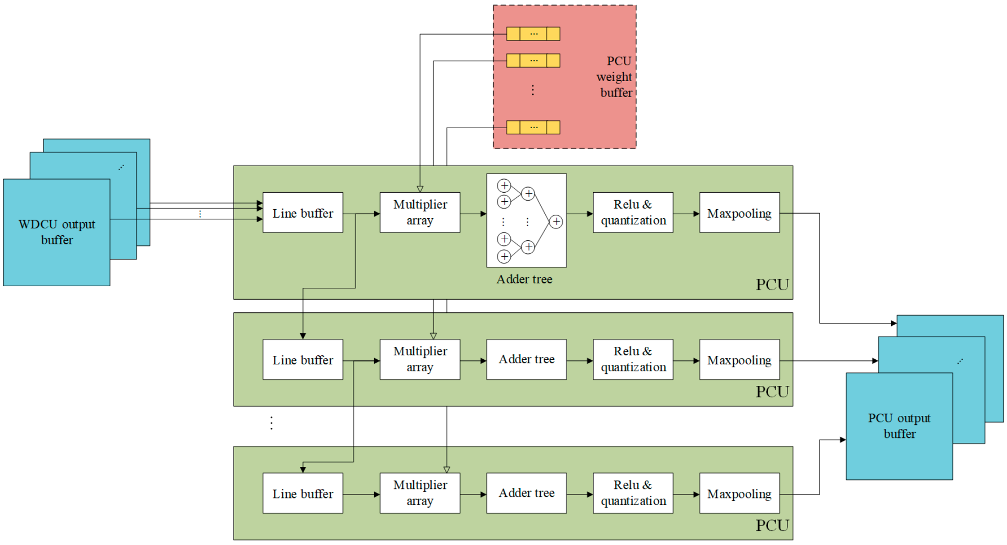

Figure S2. Design of PCU

Section S3. FCU Design

In the fully connected layer, since the weight sharing and data are not reused, a compromise solution used in [2,3] were adopted in our design. As shown in Figure S3, the FC layer contains input with 290 dimensions, the output with 7 dimensions, and a weight matrix with a 290×7 size. We used a structural design similar to PCU to optimize the FC layer for parallel operating. Since there are 10 PCU output buffers, the input matrix is set to 29×10, and the parallel input is divided into small scale vectors with 5 dimensions as shown in Figure S3. The FCU comprises line buffer, multiplier array unit, adder tree unit, accumulators unit, and quantization unit, as shown in Figure S4. Each 1×5 small scale vector is sent to the multiplier array unit through the line buffer for multiplication and is simultaneously passed to the next FCU line buffer unit. The output of the adder tree unit will be accumulated in the accumulator unit. The quantization unit quantizes the output, and finally, the max unit conducts the comparison of the 7-dimension output that the comparison result will be sent to the PS block.

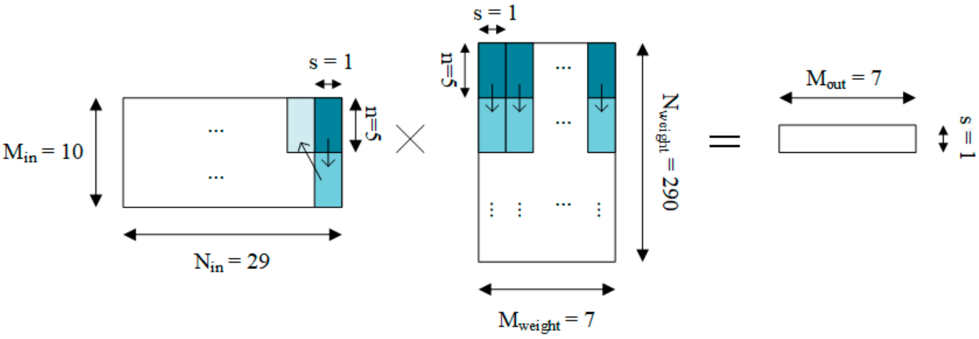

Figure S3. The weight windows in the weight matrix shifts parallel and vertically

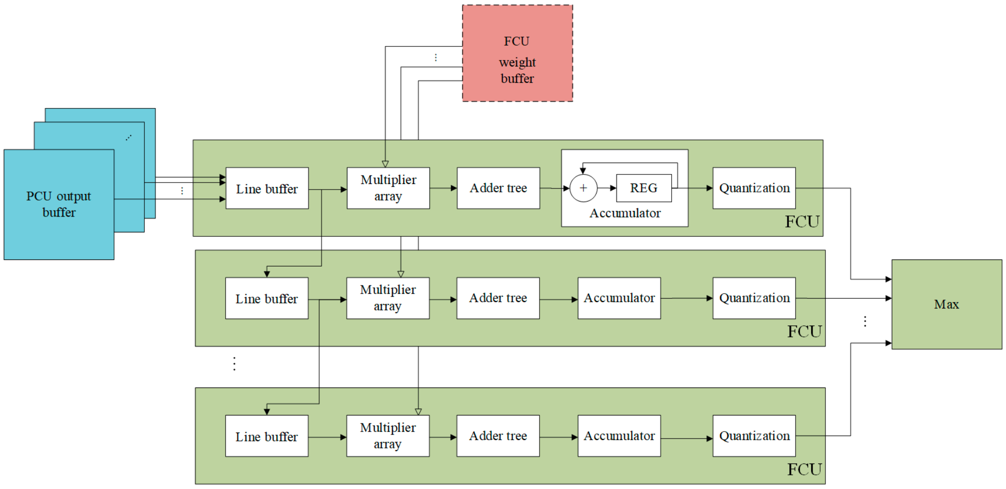

Figure S4. Design of FCU

Reference

1. Lavin, A.; Gray, S. Fast Algorithms for Convolutional Neural Networks. In Proceedings of the 2016 IEEE Conference on Computer Vision and Pattern Recognition (CVPR); IEEE: Las Vegas, NV, USA, June 2016; pp. 4013–4021.
2. Liang, Y.; Lu, L.; Xiao, Q.; Yan, S. Evaluating Fast Algorithms for Convolutional Neural Networks on FPGAs. *IEEE Trans. Comput.-Aided Des. Integr. Circuits Syst.* 2020, *39*, 857–870.
3. Huimin Li; Xitian Fan; Li Jiao; Wei Cao; Xuegong Zhou; Lingli Wang A High Performance FPGA-Based Accelerator for Large-Scale Convolutional Neural Networks. In Proceedings of the 2016 26th International Conference on Field Programmable Logic and Applications (FPL); IEEE: Lausanne, Switzerland, August 2016; pp. 1–9.
